# Physical activity levels and influencing factors among children with leukemia during chemotherapy: a cross-sectional study

**DOI:** 10.3389/fped.2026.1801600

**Published:** 2026-04-23

**Authors:** Yuchen Chu, Rui Zhang, Chen Sun, Wanhua Ren, Peiying Wang, Tianxin Liu, Yunfeng Li

**Affiliations:** 1 School of Nursing, Shandong First Medical University, Jinan, China; 2 School of Nursing, Shandong Second Medical University, Weifang, China; 3 Department of Pediatric Hematology, The First Affiliated Hospital of Shandong First Medical University, Jinan, China; 4 Department of Pediatric Hematology and Endocrinology, Provincial Hospital Affiliated to Shandong First Medical University, Jinan, China; 5 Quality Control Office, The First Affiliated Hospital of Shandong First Medical University, Jinan, China

**Keywords:** chemotherapy, children, influencing factors, leukemia, physical activity

## Abstract

**Background:**

Physical activity is an important intervention method for promoting the physical and mental recovery of children with leukemia. How to maintain or enhance the physical activity level of these children during treatment remains a significant issue in clinical rehabilitation and health management. This study aimed to investigate factors influencing physical activity among pediatric leukemia patients during chemotherapy, providing a reference for clinical healthcare providers to develop targeted physical activity intervention programs.

**Methods:**

This cross-sectional study employed convenience sampling to recruit 205 pediatric leukemia patients undergoing chemotherapy in the pediatric hematology departments of two tertiary hospitals in Shandong Province, China, from October 2024 to February 2025. Questionnaire data were analyzed using multiple linear regression to identify influencing factors.

**Results:**

This cross-sectional study included 205 pediatric leukemia patients undergoing chemotherapy (mean age 9.47 ± 3.16 years; 57.6% male). At enrollment, 30.7% were in induction, 48.3% in consolidation, and 20.9% in maintenance. LSI scores ranged from 0 to 86, with a median of 18.00 (P25 = 9.00, P75 = 30.00). Low-intensity activities were reported by 179 participants (87.3%), moderate-intensity activities by 60 participants (29.3%), and vigorous-intensity activities by 10 participants (4.9%). Based on the Godin classification (moderate-to-vigorous LSI ≥24), 19 participants (9.3%) were classified as active. Multiple linear regression analysis identified chemotherapy phase, symptom burden, maternal support, and exercise self-efficacy as significant independent predictors of physical activity levels (*P* < 0.001), collectively explaining 47.7% of the variance.

**Conclusion:**

Pediatric leukemia patients during chemotherapy demonstrate low physical activity levels, primarily influenced by chemotherapy phase, symptom burden, maternal support, and exercise self-efficacy. Clinical interventions should adopt a phased approach aligned with treatment intensity, emphasize family engagement, and incorporate individualized goal-setting to enhance children's physical activity participation and overall quality of life.

## 1 Introduction

Leukemia is a malignant clonal disease of hematopoietic stem cells. It represents approximately one-third of all childhood cancers, making it one of the most prevalent pediatric malignancies (1). With advancements in treatment systems and the increasing precision and individualization of therapeutic regimens, the overall survival rate of pediatric leukemia patients has significantly improved. In many high-income countries, the five-year survival rate for children with acute lymphoblastic leukemia (ALL) now exceeds 80% (2). Chemotherapy remains the main method for treating childhood leukemia in clinical practice. It primarily targets rapidly dividing leukemic cells by exploiting their increased sensitivity to chemotherapeutic agents, thereby inducing cell death and inhibiting tumor growth. However, these drugs can also affect normal and healthy cells because they lack complete selectivity. As a result, chemotherapy may lead to declines in children's neurological and motor functions (3), as well as a series of adverse reactions such as pain, fatigue, anxiety, and depression, thereby affecting their quality of life (4).

Physical activity (5) is defined as any bodily movement produced by skeletal muscles that results in energy expenditure, constituting a fundamental determinant of physical health. Accumulating evidence (6, 7) demonstrates that regular physical activity can effectively enhance muscle strength, cardiorespiratory fitness, and overall physical function in childhood cancer survivors while alleviating cancer-related fatigue. Moreover, it contributes to the mitigation of emotional distress such as anxiety and depression. Research (8) also indicates that acute exercise may enhance natural killer cell function, thereby optimizing the immune microenvironment in pediatric patients and augmenting their anti-inflammatory and anti-tumor capacities. This suggests that physical activity could serve as a potential adjunct to immunotherapy to enhance therapeutic efficacy. International guidelines (9) and consensus (10) unanimously emphasize that children with leukemia should start physical activities as early as possible after diagnosis and integrate exercise rehabilitation throughout the entire treatment process and subsequent follow-up stages. Early intervention can effectively prevent or alleviate muscle atrophy caused by long-term bed rest and various side effects of treatment. Individualized adjustments of exercise under medical monitoring are not only safe and feasible but may also enhance the tolerance of children to treatment. Therefore, engaging in moderate physical activities under medical monitoring has become a safe and necessary part of the comprehensive treatment of leukemia.

Physical activity levels among pediatric leukemia patients are typically low during treatment (11–13). If unaddressed, this inactivity can exacerbate fatigue and increase the risks of muscle atrophy, cardiovascular complications, and other adverse outcomes, potentially impairing recovery and long-term prognosis. To mitigate these risks, it is essential to assess the current status of physical activity among these patients, identify key determinants, and develop evidence-based intervention strategies to promote patient engagement. At present, most studies on physical activity of children with leukemia focus on the rehabilitation period or the long-term follow-up stage. The understanding of the physical activity status during chemotherapy and its influencing mechanisms is still relatively limited. Family-Centered Care (FCC) (14) recognizes the family as the primary source of strength and support for children, particularly during illness and treatment. This framework posits that pediatric health behaviors are shaped not only by individual characteristics but also by family dynamics, parental support, and the home environment. Guided by this framework, the present study examines individual and family-level factors influencing physical activity among pediatric leukemia patients undergoing chemotherapy. This study aims to identify factors influencing physical activity in children with leukemia during chemotherapy, thus providing clinical healthcare providers with evidence to develop targeted interventions.

## 2 Methods

### 2.1 Study design

This cross-sectional survey study was conducted among pediatric leukemia patients undergoing chemotherapy at two tertiary hospitals in Shandong Province, China, between October 2024 and February 2025.

### 2.2 Participants

Children undergoing chemotherapy were recruited through convenience sampling from the pediatric hematology departments of two tertiary hospitals. Both institutions are nationally designated hospitals for pediatric hematological diseases. They serve as major referral centers in the region, receiving patients from both urban and rural areas across Shandong Province. Eligible participants were children aged 6–17 years with a clinical diagnosis of leukemia who were currently receiving chemotherapy and had completed at least one treatment cycle. Written informed consent was required from both the patient and at least one parent. Patients were excluded if they had concurrent malignancies, severe comorbidities or vital organ impairment (heart, brain, or lungs), prior hematopoietic stem cell transplantation or radiotherapy, or contraindications to physical activity (body temperature >38 °C, platelet count <20 × 10⁹/L, active bleeding, or limited joint mobility due to osteonecrosis, arthritis, synovitis, or neurotoxicity). This study has been approved by the university ethics committee (Approval No. R202410240368).

### 2.3 Measures

Based on the conceptual framework derived from FCC theory, predictor variables were categorized into two dimensions: individual-level factors (such as the child's sociodemographic characteristics, disease-related factors, symptom burden, and self-efficacy) and family-level factors (such as family structure, economic status, and parental support for physical activity).

#### 2.3.1 General information questionnaire

A self-designed questionnaire was administered to collect sociodemographic and disease-related information. The child's sociodemographic data included gender, age, residence, status as an only child. Disease-related information included the child's diagnosis, chemotherapy phase, risk level, history of other diseases, method of medical expense payment, medical insurance reimbursement rate, and other relevant details. Family structure and economic status included primary caregiver role, education level of the primary caregiver, occupation of the primary caregiver, marital status of the caregiver, and monthly household income per capita.

#### 2.3.2 Godin leisure time exercise questionnaire (GLTEQ)

Developed by Godin (15) to assess an individual's low, moderate, and high intensity physical activity over the past 7 days. The questionnaire seeks information on the number of times one engages in mild (minimal effort), moderate (not exhausting), and strenuous (heart beats rapidly) physical activity of at least 15-min. The weekly frequency of mild, moderate, and strenuous activities was multiplied by the corresponding metabolic equivalent (MET) values (3, 5, and 9 METs, respectively), and summed to obtain the Leisure Score Index (LSI). The calculation formula is as follows: LSI = (Mild frequency×3) + (Moderate frequency×5) + (Strenuous frequency×9). To facilitate clinical interpretation, participants' physical activity levels were categorized based on the validation study by Amireault and Godin (16). This classification system is based solely on the moderate-to-vigorous LSI. Active: Moderate-to-vigorous LSI ≥ 24. Insufficiently Active: Moderate-to-vigorous LSI ≤23. The GLTEQ has been widely used in oncology research (17), has demonstrated acceptable reliability and validity in pediatric populations (18), and has been successfully applied in childhood leukemia survivors (19, 20) as well as in Chinese childhood cancer populations (21). Although the participants in this study are children undergoing active chemotherapy, the 7-day recall period of the GLTEQ aligns well with chemotherapy cycles, and the questionnaire's brevity and low burden make it suitable for children who may experience fatigue. Following the strategy of Tillmann et al. (20), children <10 years of age were assessed by parental proxy report, while children ≥10 years self-reported with parental assistance.

#### 2.3.3 Activity support scale for multiple groups (ACTS-MG)

Lampard et al. (22) revised the Activity Support Scale (ACTS) to develop a child-reported version suitable for multiple ethnic groups, the Activity Support Scale for Multiple Groups (ACTS-MG), which assesses children's perceptions of parental physical activity support behaviors. This scale consists of two scales, the Mother Version and the Father Version, each with nine items on a 4-point Likert scale. In this study, the Cronbach's *α* coefficient was 0.751 for the mother version and 0.720 for the father version.

#### 2.3.4 Memorial symptom assessment scale 10–18 (MSAS 10–18)

The scale was developed by Collins et al. (23) based on the adult version, it assesses symptom experiences in pediatric cancer patients aged 10–18. After consulting the original authors, it was determined that the scale can also be used to evaluate symptom experiences in children under 10 with cancer. The scale comprises 31 items. Items 1–23 assess symptoms based on occurrence, frequency, severity, and distress. Items 24–31 evaluate symptoms based on occurrence, severity, and distress. Symptom frequency and severity are measured using a 4-point Likert scale, while distress is assessed using a 5-point Likert scale. The symptom score for each item is the average of the frequency, severity, and distress scores. A higher symptom score indicates a greater overall impact of the symptom on the child. The Cronbach's *α* coefficient for this scale in this study was 0.811.

#### 2.3.5 Exercise self-efficacy scale (ESES)

The Exercise Self-Efficacy Scale was developed by Bandura (24) to assess respondents' confidence in adhering to regular physical exercise or activity across various challenging situations. It comprises 18 items, each scored on a 0–100 scale. A score of 100 indicates complete confidence, 50 indicates moderate confidence, and 0 indicates no confidence. The total physical self-efficacy score is the average of all item scores. Higher scores indicate greater physical self-efficacy. In this study, the Cronbach's *α* coefficient was 0.927.

### 2.4 Data collection

Research team members, who received standardized training, conducted face-to-face interviews with children. Interviews were scheduled at appropriate times, avoiding periods of severe weakness following chemotherapy, mealtimes, or when patients were irritable or resistant. After obtaining informed consent from the child and their family, the interviewer explained the study's purpose and the method for completing the questionnaire. For children under 10 years old, the interviewer assisted with responses alongside the parent. The interviewer used simple, understandable language to explain the questionnaire content or demonstrate its use, with parental assistance to ensure the child understood the questions. Questionnaires were collected on-site, checked for omissions, and cross-referenced with medical records to verify patient details. Missing or erroneous entries were corrected after confirmation with the patient or family. Two reviewers performed data entry to ensure accuracy.

### 2.5 Data analysis

Statistical analysis was performed using SPSS 26.0 software. LSI scores showed a right-skewed distribution. Therefore, medians and interquartile ranges were used for descriptive statistics. Qualitative data were represented by frequency and composition ratio (%). Nonparametric tests were used for univariate analysis, Spearman correlation analysis for correlation studies, and multiple linear regression analysis for multivariate analysis. All variables were included in the regression analysis as independent predictors.

## 3 Results

### 3.1 General characteristics of study subjects and univariate analysis results

We initially enrolled 226 children who met the basic eligibility criteria. Parents of 16 children declined, and 210 questionnaires were distributed. After data collection and verification, 5 questionnaires were excluded due to extensive missing data (*n* = 2) or patterned responses (*n* = 3), yielding 205 valid responses with an effective response rate of 97.62%. All 205 included cases had complete data with no missing values. The baseline characteristics of the 205 enrolled children are presented in Table 1. There were slightly more males (*n* = 128) than females (*n* = 77), with a mean age of 9.47 ± 3.16 years. The majority of children were diagnosed with acute lymphoblastic leukemia (*n* = 162, 79.0%). 118 participants (57.6%) resided in urban areas and 87 (42.4%) in rural areas at the time of enrollment. Univariate analysis revealed that disease diagnosis, chemotherapy phase, risk level, chemotherapy status within the past 7 days, and presence of other medical conditions were statistically significant factors influencing physical activity scores (*P* < 0.05), as shown in Table 1.

**Table 1:** General characteristics and univariate analysis of physical activity.

Item	Number (Percentage, %)	LSI Median (P_25_, P_75_)	Test Statistic Z/H	*p*-value
Gender			−0.129	0.897
Male	128 (62.4%)	18.00 (9.00, 30.00)		
Female	77 (37.6%)	18.00 (9.00, 31.00)		
Age			−1.297	0.195
6–12	120 (58.5%)	18.00 (9.00, 31.00)		
13–17	85 (41.5%)	15.00 (9.00, 21.00)		
Place of residence			−1.573	0.116
Urban	118 (57.6%)	15.00 (6.00, 30.00)		
Rural	87 (42.4%)	21.00 (9.00, 31.00)		
Status as an only child			−1.106	0.269
Yes	50 (24.4%)	15.00 (6.00, 30.00)		
No	155 (75.6%)	18.00 (9.00, 31.00)		
Primary caregiver role			−0.305	0.760
Father	37 (18.0%)	18.00 (7.50, 30.00)		
Mother	168 (82%)	18.00 (9.00, 30.75)		
Education level of the primary caregiver			0.482	0.923
Junior high school or below	65 (31.7%)	18.00 (9.00, 31.00)		
High school or vocational school	72 (35.1%)	18.00 (9.00, 30.00)		
College or university	63 (30.7%)	18.00 (9.00, 30.00)		
Graduate	5 (2.4%)	15.00 (12.00, 35.50)		
Occupation of the primary caregiver			−0.902	0.367
Employed	107 (52.2%)	18.00 (9.00, 31.00)		
Unemployed	98 (47.8%)	16.50 (9.00, 30.00)		
Marital status of the caregiver			−0.650	0.515
Married	200 (97.6%)	18.00 (9.00, 30.00)		
Divorced	5 (2.4%)	21.00 (9.50, 46.00)		
Monthly household income per capita(Yuan)			1.714	0.634
<1,000	10 (4.9%)	16.50 (8.25, 33.00)		
1,000–3,000	38 (18.5%)	15.00 (6.00, 31.00)		
3,000–5,000	109 (53.2%)	18.00 (9.00, 30.00)		
>5,000	48 (23.4%)	18.00 (12.75, 31.00)		
Disease Diagnosis			−2.544	0.011
AML	43 (21.0%)	12.00 (6.00, 30.00)		
ALL	162 (79%)	18.00 (9.00, 31.00)		
Chemotherapy Phase			52.063	<0.001
Induction	63 (30.7%)	9.00 (3.00, 19.00)		
Consolidation	99 (48.3%)	15.00 (9.00, 24.00)		
Maintenance	43 (20.9%)	36.00 (27.00, 51.00)		
Risk Level			27.503	<0.001
Low	58 (28.3%)	28.50 (18.00, 41.00)		
Moderate	132 (64.4%)	15.00 (9.00, 25.00)		
High	15 (7.3%)	6.00 (0.00, 18.00)		
Chemotherapy in the Past Seven Days			−3.201	0.001
Yes	170 (82.9%)	15.00 (9.00, 27.00)		
No	35 (17.1%)	31.00 (12.00, 36.00)		
Other medical history			−2.424	0.015
There is	4 (2.0%)	0.00 (0.00,11.25)		
None	201 (98.0%)	18.50 (9.00, 30.50)		
Medical Expense Payment Method			0.545	0.909
Municipal Resident Medical Insurance	33 (16.1%)	15.00 (7.50, 31.00)		
Intra-provincial cross-regional medical insurance	162 (79.0%)	18.00 (9.00, 30.25)		
Commercial Insurance and Provincial Cross-Regional Medical Insurance	6 (2.9%)	19.50 (15.75, 26.25)		
Out-of-pocket	4 (2.0%)	15.50 (9.25, 22.50)		
Medical insurance reimbursement rate			0.085	0.959
30%–50%	162 (79.0%)	18.00 (8.25, 30.00)		
50%–80%	39 (19.0%)	18.00 (9.00, 31.00)		
0 (self-funded)	4 (2.0%)	15.50 (9.25, 22.50)		

ALL, Acute Lymphoblastic Leukemia; AML, Acute Myeloid Leukemia.

### 3.2 Physical activity levels

The physical activity status of children with leukemia is shown in Table 2. Physical activity was assessed using the LSI, with higher scores indicating greater physical activity levels. LSI scores showed a right-skewed distribution (skewness = 1.243, kurtosis = 1.513), with a median of 18.00 (P_25_ = 9.00, P_75_ = 30.00) and a mean of 21.25 ± 18.00 (range: 0–86). Overall, patients exhibited low physical activity levels with an extremely skewed intensity distribution: predominantly low-intensity activities (87.32% participation rate), while moderate and vigorous activities were infrequent (29.27% and 4.88% participation rates, respectively), with median scores of 0 for both. According to the classification criteria proposed by Amireault and Godin (16) (moderate-to-vigorous LSI ≥24 defined as active), only 19 children (9.3%) in this study met the active level.

**Table 2:** Current physical activity Status of children with leukemia.

Activity intensity level	LSI Range	LSI Median (P_25_–P_75_)	Participation Rate (%)
Low-intensity activity (MET)	0–45	15 (9, 21)	87.32
Moderate-intensity activity (MET)	0–35	0 (0, 10)	29.27
High-intensity activity (MET)	0–27	0 (0, 0)	4.88
Total Activity Score	0–86	18 (9, 30)	87.32

MET, Metabolic Equivalent of Task; a unit used to measure the energy expenditure of physical activity, where 1 MET is equivalent to the metabolic rate at rest. LSI, the Leisure Score Index.

### 3.3 Correlation analysis

As shown in Table 3, children's symptoms were negatively correlated with physical activity scores, while parental support and self-efficacy were positively associated with physical activity scores.

**Table 3:** Correlation analysis of physical activity(LSI) in pediatric leukemia patients.

Variable	LSI	Symptoms	Maternal support	Parental support	Exercise self-efficacy
LSI	1				
Symptoms	−0.587**	1			
Maternal Support	0.471**	−0.250**	1		
Parental Support	0.295**	−0.262**	0.646**	1	
Exercise Self-Efficacy	0.393**	−0.297**	0.243**	0.229**	1

** *P* < 0.01. LSI, the Leisure Score Index.

### 3.4 Multiple linear regression analysis

Multivariate linear regression analysis was conducted using the child's physical activity score as the dependent variable and the variables showing statistically significant differences in univariate analysis and those correlated with the child's physical activity as independent variables. Variable coding was as follows: Disease Diagnosis: Acute Myeloid Leukemia = 1, Acute Lymphoblastic Leukemia = 2; Chemotherapy Phase: Induction phase(Z1 = 0, Z2 = 0, Z3 = 0), Consolidation phase(Z1 = 0, Z2 = 1, Z3 = 0), Maintenance phase(Z1 = 0, Z2 = 0, Z3 = 1); Risk Level: Low risk(Z1 = 0, Z2 = 0, Z3 = 0), Intermediate risk(Z1 = 0, Z2 = 1, Z3 = 0), High risk(Z1 = 0, Z2 = 0, Z3 = 1); Chemotherapy in the past 7 days: Yes = 1, No = 2; Other medical history: Yes = 1, No = 2. Symptoms, maternal support, paternal support, and exercise self-efficacy were entered as raw values. Regression diagnostics indicated that the assumptions of residual linearity and normality were reasonably met. Although the residual plot suggested mild heteroscedasticity, bootstrap resampling (1,000 samples) confirmed the stability of all significant predictors. The variance inflation factor (VIF) values for all variables ranged from 1.079 to 2.546, all well below the commonly accepted threshold of 5. This indicates that no severe multicollinearity was present. As shown in Table 4, chemotherapy phase, symptom burden, maternal support, and self-efficacy were significantly associated with physical activity. Compared to the induction phase, the consolidation phase was not significant, whereas the maintenance phase showed a positive association. Disease diagnosis (ALL vs. AML) was not a significant predictor. The standardized regression coefficients for these significant predictors are illustrated in Supplementary Figure S1. The distribution of physical activity across chemotherapy phases is shown in Supplementary Figure S2, and the results of the stratified regression analysis are presented in Table 5.

**Table 4:** Multivariate linear regression analysis results for factors influencing physical activity in pediatric leukemia patients.

Variable	B	Standard Error	Beta	*t*	*P*	95% CI	Bootstrap BCa 95% CI	Collinearity Statistics Tolerance	VIF
(Constant)	−22.888	16.395		−1.396	0.164	(−55.224,9.447)	(−48.953,1.968)		
Disease Diagnosis	1.921	2.397	0.044	0.802	0.424	(−2.806,6.649)	(−3.738,7.090)	0.868	1.152
Chemotherapy in the Past Seven Days	0.744	2.606	0.016	0.286	0.775	(−4.396,5.885)	(−6.063,7.396)	0.86	1.163
Other medical history	−0.631	6.828	−0.005	−0.092	0.926	(−14.098,12.836)	(−6.230,5.393)	0.927	1.079
Consolidation phase	1.457	2.245	0.041	0.649	0.517	(−2.970,5.885)	(−2.267,5.174)	0.657	1.522
Maintenance phase	9.726	3.452	0.220	2.818	0.005	(2.918,16.533)	(2.614,16.901)	0.419	2.388
Medium risk	1.165	2.431	0.031	0.479	0.632	(−3.630,5.960)	(−3.973,6.420)	0.61	1.639
High risk	1.943	4.827	0.028	0.403	0.688	(−7.577,11.463)	(−5.772,9.660)	0.523	1.911
Symptoms	−23.764	5.94	−0.323	−4.001	<0.001	(−35.479,−12.049)	(−33.558,−13.162)	0.393	2.546
Maternal Support	2.355	0.449	0.375	5.251	<0.001	(1.470,3.24)	(1.324,3.330)	0.504	1.985
Parental Support	−0.767	0.468	−0.113	−1.64	0.103	(−1.690,0.155)	(−1.736,0.153)	0.543	1.843
Exercise Self-Efficacy	0.299	0.082	0.204	3.643	<0.001	(0.137,0.46)	(0.150,0.452)	0.819	1.221

B: Unstandardized Coefficient. Beta: Standardized Coefficient. CI: Confidence Interval. R^2^ = 0.505, adjusted R2 = 0.477. *F* = 17.913, *P* < 0.001.

**Table 5:** Results of stratified linear regression analysis.

Model	Variable	B	Unstandardized Coefficient	Beta	*t*	*P*	95% CI	Collinearity Statistics Tolerance	VIF
1	(Constant)	−9.83	16.499		−0.596	0.552	(−42.366,22.707)		
	Disease Diagnosis	2.128	2.741	0.048	0.776	0.438	(−3.277,7.533)	0.899	1.113
	Chemotherapy in the Past Seven Days	5.252	2.964	0.11	1.772	0.078	(−0.594,11.098)	0.899	1.112
	Other medical history	9.901	7.719	0.076	1.283	0.201	(−5.322,25.124)	0.982	1.019
	Consolidation phase	4.509	2.534	0.125	1.779	0.077	(−0.489,9.507)	0.698	1.433
	Maintenance phase	19.646	3.469	0.445	5.664	<0.001	(12.806,26.486)	0.561	1.782
	Medium risk	−6.062	2.597	−0.162	−2.334	0.021	(−11.184,−0.94)	0.723	1.382
	High risk	−12.024	4.63	−0.174	−2.597	0.01	(−21.155,−2.894)	0.77	1.299
2	(Constant)	9.835	16.671		0.59	0.556	(−23.042,42.712)		
	Disease Diagnosis	3.67	2.672	0.083	1.373	0.171	(−1.6,8.94)	0.88	1.137
	Chemotherapy in the Past Seven Days	3.522	2.893	0.074	1.218	0.225	(−2.183,9.227)	0.879	1.138
	Other medical history	4.382	7.575	0.034	0.578	0.564	(−10.557,19.322)	0.948	1.054
	Consolidation phase	2.882	2.479	0.08	1.162	0.246	(−2.007,7.771)	0.679	1.474
	Maintenance phase	12.429	3.81	0.282	3.263	0.001	(4.916,19.942)	0.433	2.31
	Medium risk	−2.443	2.667	−0.065	−0.916	0.361	(−7.702,2.817)	0.639	1.566
	High risk	−1.052	5.255	−0.015	−0.2	0.842	(−11.415,9.311)	0.556	1.798
	Symptoms	−25.33	6.392	−0.345	−3.963	<0.001	(−37.935,−12.724)	0.427	2.341
3	(Constant)	−9.156	17.977		−0.509	0.611	(−44.61,26.297)		
	Disease Diagnosis	3.083	2.643	0.07	1.166	0.245	(−2.13,8.296)	0.873	1.145
	Chemotherapy in the Past Seven Days	3.26	2.853	0.068	1.143	0.255	(−2.366,8.886)	0.878	1.139
	Other medical history	2.429	7.504	0.019	0.324	0.747	(−12.37,17.228)	0.939	1.065
	Consolidation phase	3.086	2.445	0.086	1.262	0.208	(−1.735,7.907)	0.678	1.475
	Maintenance phase	12.819	3.758	0.291	3.411	0.001	(5.408,20.229)	0.432	2.314
	Medium risk	−1.893	2.637	−0.05	−0.718	0.474	(−7.094,3.307)	0.634	1.576
	High risk	−0.161	5.19	−0.002	−0.031	0.975	(−10.397,10.076)	0.554	1.806
	Symptoms	−22.916	6.367	−0.312	−3.599	<0.001	(−35.474,−10.358)	0.418	2.391
	Paternal support	1.046	0.402	0.154	2.604	0.01	(0.254,1.838)	0.9	1.111
4	(Constant)	−16.771	16.816		−0.997	0.32	(−49.937,16.395)		
	Disease Diagnosis	2.055	2.471	0.047	0.831	0.407	(−2.819,6.929)	0.868	1.152
	Chemotherapy in the Past Seven Days	1.53	2.678	0.032	0.571	0.568	(−3.752,6.812)	0.866	1.155
	Other medical history	−1.65	7.035	−0.013	−0.235	0.815	(−15.525,12.224)	0.929	1.077
	Consolidation phase	0.984	2.311	0.027	0.426	0.671	(−3.573,5.542)	0.659	1.517
	Maintenance phase	9.396	3.558	0.213	2.641	0.009	(2.379,16.413)	0.419	2.387
	Medium risk	0.727	2.504	0.019	0.29	0.772	(−4.211,5.665)	0.612	1.635
	High risk	4.659	4.917	0.068	0.947	0.345	(−5.039,14.357)	0.536	1.865
	Symptoms	−27.983	6.007	−0.381	−4.658	<0.001	(−39.831,−16.136)	0.408	2.449
	Paternal support	−0.611	0.48	−0.09	−1.272	0.205	(−1.559,0.336)	0.547	1.828
	Maternal support	2.533	0.46	0.403	5.509	<0.001	(1.626,3.44)	0.51	1.962
5	(Constant)	−22.888	16.395		−1.396	0.164	(−55.224,9.447)		
	Disease Diagnosis	1.921	2.397	0.044	0.802	0.424	(−2.806,6.649)	0.868	1.152
	Chemotherapy in the Past Seven Days	0.744	2.606	0.016	0.286	0.775	(−4.396,5.885)	0.86	1.163
	Other medical history	−0.631	6.828	−0.005	−0.092	0.926	(−14.098,12.836)	0.927	1.079
	Consolidation phase	1.457	2.245	0.041	0.649	0.517	(−2.97,5.885)	0.657	1.522
	Maintenance phase	9.726	3.452	0.22	2.818	0.005	(2.918,16.533)	0.419	2.388
	Medium risk	1.165	2.431	0.031	0.479	0.632	(−3.63,5.96)	0.61	1.639
	High risk	1.943	4.827	0.028	0.403	0.688	(−7.577,11.463)	0.523	1.911
	Symptoms	−23.764	5.94	−0.323	−4.001	<0.001	(−35.479,−12.049)	0.393	2.546
	Paternal support	−0.767	0.468	−0.113	−1.64	0.103	(−1.69,0.155)	0.543	1.843
	Maternal support	2.355	0.449	0.375	5.251	<0.001	(1.47,3.24)	0.504	1.985
	Exercise Self-Efficacy	0.299	0.082	0.204	3.643	<0.001	(0.137,0.46)	0.819	1.221

B: Unstandardized Coefficient. Beta: Standardized Coefficient. CI, Confidence Interval.

According to Green (25), a minimum sample size of *N* ≥ 104+*k* is recommended for testing individual predictors in multiple regression, where *k* is the number of predictors(excluding the intercept). With *k* = 11, at least 115 participants are required. Our sample of 205 participants exceeds this threshold, ensuring adequate statistical power for stable parameter estimation. A *post-hoc* power analysis further confirmed that the achieved power exceeded 0.99 based on the observed effect size (*R*2 = 0.505), confirming that the final sample of 205 participants was more than sufficient to detect the effects reported.

## 4 Discussion

### 4.1 Pediatric leukemia patients primarily engage in low-intensity exercise during chemotherapy, with prolonged periods of sitting

This study found that children undergoing chemotherapy for leukemia had markedly low physical activity levels, with only 9.3% meeting the active threshold (LSI ≥24). Participation rates for moderate- (29.27%) and vigorous-intensity (4.88%) activities were low, consistent with previous research (13, 26). Götte et al. (27) reported significantly reduced moderate-to-vigorous physical activity among childhood cancer survivors compared to healthy peers. The present study extends these findings by focusing on children during active treatment, for whom persistent side effects and intensive medical interventions make establishing regular activity habits even more challenging. The World Health Organization (WHO) recommends that children aged 5–17 years engage in at least 60 min of moderate-to-vigorous physical activity daily (≥420 min per week) (28). The proportion meeting the guideline would be even lower. However, it is important to recognize that both the Godin classification and the WHO guideline were developed for general healthy populations, not for children undergoing active cancer treatment. Consistent with this view, international pediatric oncology exercise guidelines (9, 29) emphasize that any level of physical activity is better than none, encouraging children to initiate movement as early as possible during treatment, even at low intensities. Children are in a critical period of growth and development, where physical activity is closely linked to skeletal formation (30) and cardiopulmonary function (31). Prolonged sedentary behavior and lack of activity not only hinder treatment and recovery but also impair normal growth and development. Evidence indicates that physical activity helps mitigate chemotherapy side effects and maintain bodily functions (32). Therefore, rather than interpreting the low proportion of children meeting the recommended standards as a failure, it should be seen as a call for individualized, clinically realistic activity goals that focus on maintaining some level of activity, breaking sedentary patterns, and gradually building activity habits in a safe and supportive manner. The significant variation in activity levels observed in our study supports a stratified approach to physical activity promotion, with goals tailored to each child's current status—ranging from cultivating basic activity awareness in sedentary children to gradually increasing intensity in those already maintaining daily routines.

### 4.2 Factors influencing physical activity in pediatric leukemia patients

The patient's physiological state is the primary factor determining their ability to participate in physical activity. During chemotherapy, particularly during the induction phase, high-intensity drug therapy causes impairment of the patient's bodily functions (3), resulting in significant symptom burden such as fatigue, pain, nausea, and vomiting. These symptoms are the main reasons hindering or terminating the patient's activities. These physiological symptoms not only directly limit activity capacity but also readily trigger psychological reactions like anxiety and low mood, further diminishing the child's willingness to engage. The relationship between symptom management and physical activity is not unidirectional but interactive. On one hand, severe symptom distress is associated with reduced activity levels; on the other, professionally guided, moderate, and individualized physical activity can effectively alleviate both physiological and psychological symptoms (33). Therefore, healthcare providers are advised to prioritize symptom management as the prerequisite and foundation for physical activity interventions. Following initial symptom control, scientifically guided moderate activity may help children maintain or increase their activity levels, suggesting a reciprocal association between symptom management and physical activity.

Children's self-efficacy is a key psychological factor influencing their willingness to engage in activities and maintain consistent behavior. In this study, children demonstrated low physical self-efficacy, particularly exhibiting insufficient confidence in their physical capabilities when feeling discomfort during exercise(16.05 ± 13.27). Unpleasant experiences during exercise and concerns about injury may be linked to avoidance behavior. Additionally, low scores on items such as “ when I have too much work to do”(25.71 ± 14.62) and “when I have other time commitments”(24.24 ± 14.79) indicate that children prioritize physical activities as secondary, readily abandoning exercise for other tasks. This phenomenon may stem from traditional activity formats lacking appeal to children, insufficient self-management skills, or inadequate recognition of exercise's importance (34, 35). Notably, children's self-efficacy may improve through intervention, as studies (36, 37) demonstrated that moderate exercise can enhance energy and mood in pediatric patients. Furthermore, the high scores in this study for the items “After recovering from an injury that caused me to stop exercising”(54.05 ± 21.60) and “After recovering from an illness that caused me to stop exercising”(58.88 ± 21.56) indicate that children still hold expectations for their future activity capacity, providing an important psychological foundation for intervention. Therefore, healthcare providers should help children develop a positive understanding of the benefits of moderate exercise within safe parameters. Designing safe and engaging activity plans can reduce negative experiences and cultivate enjoyment. Simultaneously, increased external guidance and immediate encouragement should be provided for younger children or those with weaker self-control, helping them establish regular exercise habits.

Beyond individual intrinsic factors, external support from the family also significantly influences children's physical activity. Dugan et al. (38) found that parental support is multifaceted, providing not only emotional companionship but also equipment and creating conditions for activity. Xu et al. (21) demonstrated that the more actively parents themselves engaged in moderate-to-vigorous physical activity (MVPA), the higher the likelihood of their children participating in MVPA, underscoring the role of parental modeling. The positive correlation between parental support and children's physical activity levels in this study is consistent with previous findings. However, in our analysis, maternal support played a stronger positive role, while paternal support showed a negative effect in multivariate analysis and was not statistically significant. To better understand this result, we conducted a stratified regression analysis. The results showed that after adjusting for disease-related factors and symptom burden, paternal support was significantly positively associated with physical activity. However, when maternal support was added into the model, the coefficient for paternal support shifted from positive to negative and became non-significant. The addition of self-efficacy in the final model did not substantially alter this pattern. This finding suggests that the positive effect of paternal support is relatively weak, likely mediated primarily through maternal support or family interaction processes rather than manifesting independently. We speculate that this may be related to the division of labor within the family. Under the influence of Confucianism, a distinct division of labor has historically characterized Chinese family life: mothers assume responsibility for child-rearing, whereas fathers focus on economic provision. Even when mothers are employed, they continue to shoulder the primary responsibility for childcare and household management (39). This cultural pattern is reflected in the present study, in which mothers were the primary caregivers in the majority of families (82%), which is consistent with previous research (40, 41). Although fathers reported comparable levels of supportive attitudes and restraint intentions in questionnaires, their influence may remain largely attitudinal. The actual time they spent with the child and their direct understanding of daily symptom fluctuations were both inferior to those of mothers. This suggests that healthcare providers should identify the primary caregiver in clinical practice and target them as the core educational recipient. For families where the mother is the primary caregiver, fathers should be encouraged to play a collaborative role by supporting the mother and fostering a supportive environment.

This study has confirmed that the chemotherapy stage is an independent and stable factor for predicting the physical activity level of children with leukemia. Patients in the maintenance phase had significantly higher physical activity levels than those in the induction phase, consistent with the previous study (13). The absence of a significant difference in physical activity levels between the induction and consolidation phases likely reflects the sustained relatively high intensity of consolidation therapy, during which chemotherapy-induced neurotoxicity and cardiotoxicity remain clinically relevant. During the acute chemotherapy period—encompassing both induction and consolidation—children experience marked declines in fatigue, activity tolerance, muscle strength, balance, and functional mobility (42, 43). In contrast, the maintenance phase involves substantially lower-intensity oral chemotherapy, allowing for gradual recovery of physical function and improved self-efficacy, which together support significantly higher physical activity levels. Therefore, it is recommended to establish a tiered physical activity intervention strategy aligned with chemotherapy phases. During intensive treatment, priority should be given to safety and maintaining basic function through low-intensity activities. During the maintenance phase, the focus should shift to enhancing physical capacity and social reintegration through progressive functional training and peer interactions.

## 5 Limitations

Several limitations should be acknowledged. First, the sample was drawn from a single region with a limited size, which may limit the generalizability of the findings. Second, physical activity was primarily assessed using self-reported questionnaires, which may introduce recall bias. Third, key variables—including physical activity, symptom burden, self-efficacy, and parental support—were assessed at a single time point and relied on the same respondent (child self-report, with parental assistance for younger children). This raises the possibility of common method variance, which may artificially inflate the observed associations and partly explain the relatively high explained variance. Future multicenter studies should incorporate objective measures (e.g., accelerometers) and multi-informant reports to address these limitations.

## 6 Conclusion

Physical activity levels among pediatric leukemia patients undergoing chemotherapy are influenced by a combination of multidimensional factors. Different stages of chemotherapy induce varying degrees of symptom distress, fundamentally limiting patients' activity capacity. Psychological distress and self-efficacy factors directly impact patients' willingness to engage in activities. Family support constitutes a critical external environmental factor. This suggests that healthcare providers should prioritize these factors and adopt phased, individualized strategies to enhance patients' activity levels and quality of life.

## Data Availability

The raw data supporting the conclusions of this article will be made available by the authors, without undue reservation.
