# Supplementary material for: Physical activity levels and influencing factors among children with leukemia during chemotherapy: a cross-sectional study
Source: Front Pediatr. 2026 Apr 23;14:1801600. doi: 10.3389/fped.2026.1801600 (PMC13149412; doi:10.3389/fped.2026.1801600)
Supplement: Supplementary file 1 [file Supplementaryfile1.docx]

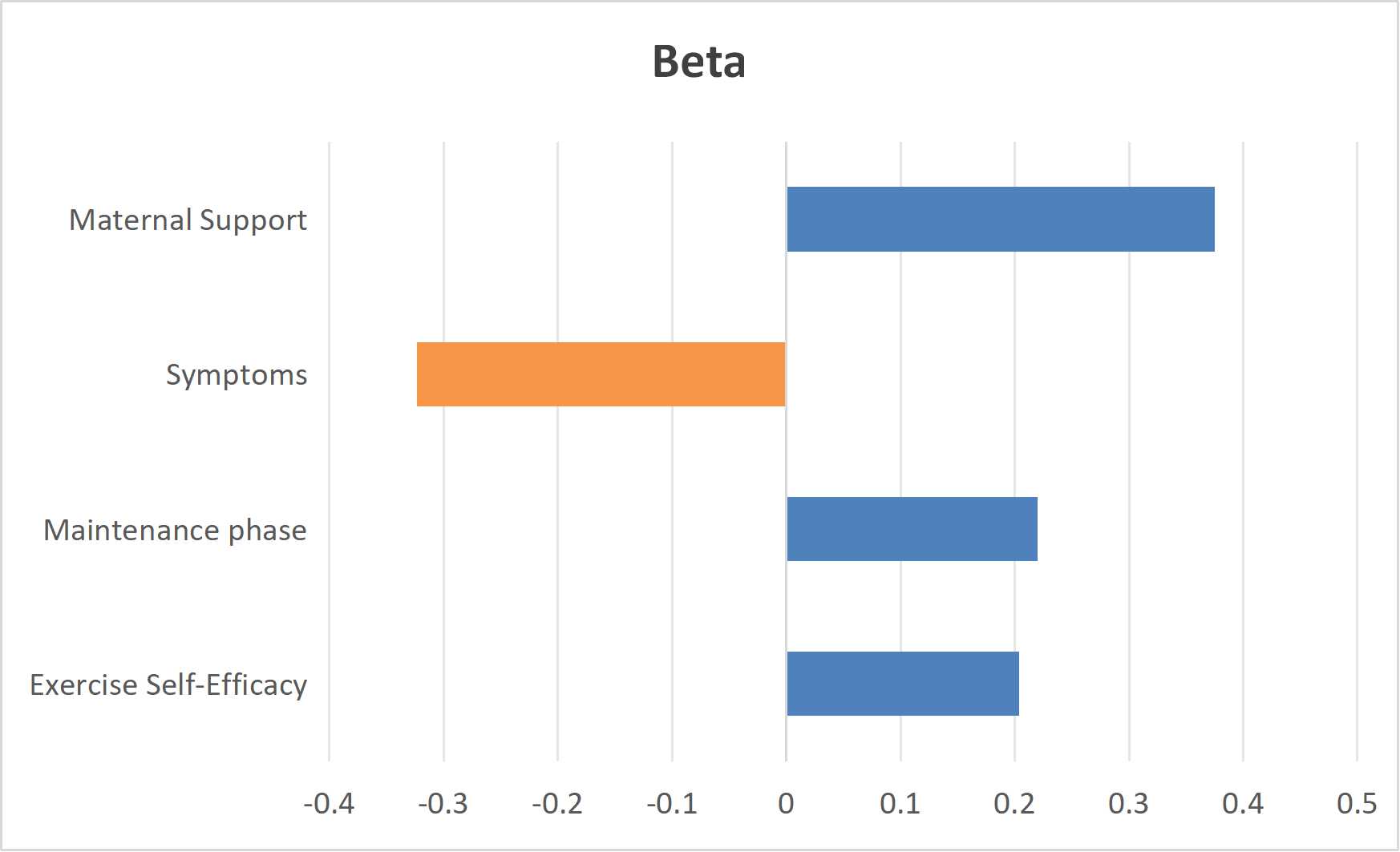


**Supplementary Figure S1.Standardized regression coefficients (β) for significant predictors of physical activity.**

Positive coefficients (blue) indicate positive associations; negative coefficients (red) indicate negative associations.


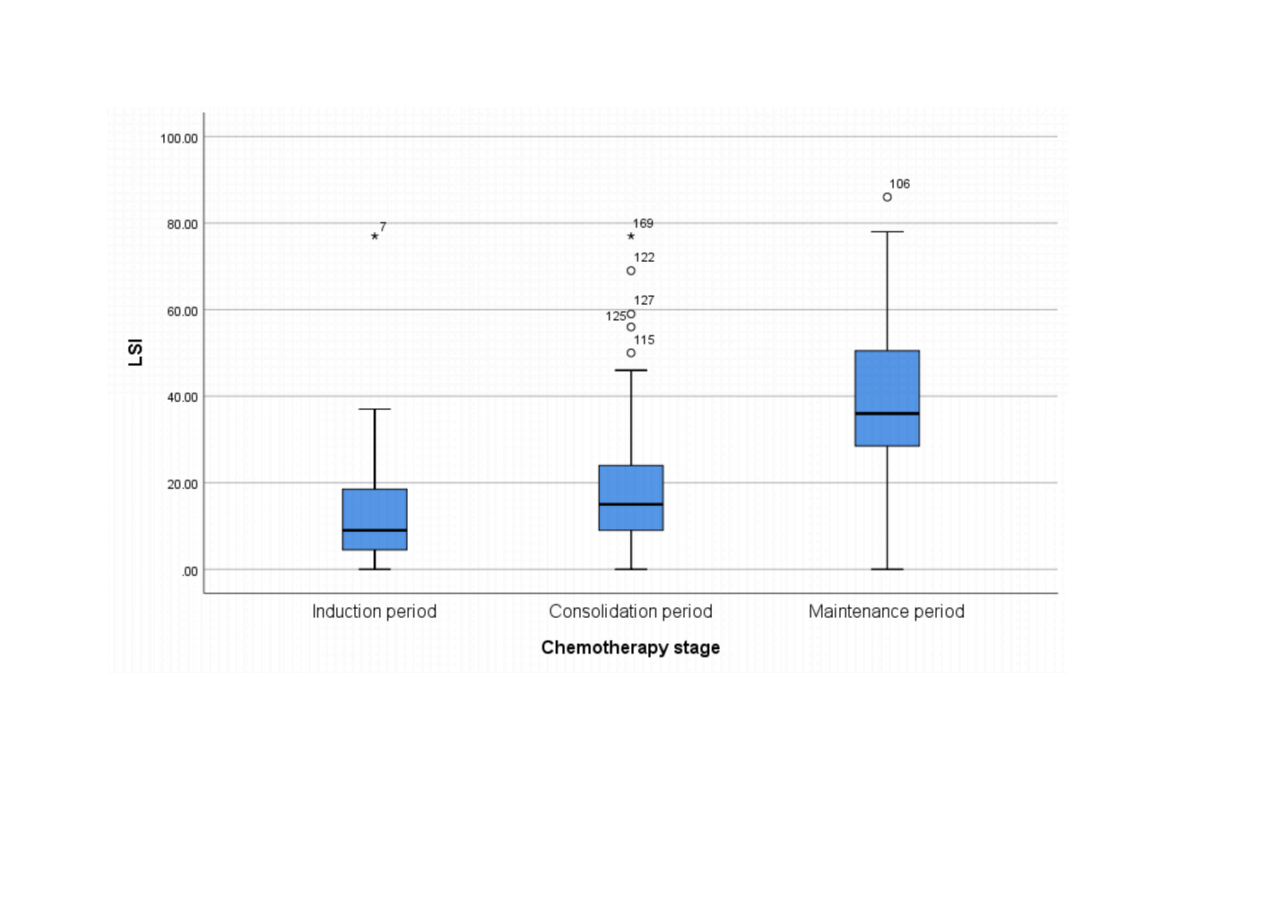


**Supplementary Figure S2. Physical activity levels across chemotherapy phases.**

Box plots show the median (horizontal line), interquartile range (box), and range (whiskers) of Leisure Score Index (LSI) scores for children in the induction (n = 63), consolidation (n = 99), and maintenance (n = 43) phases. Circles (○) indicate outliers, and asterisks (★) indicate extreme values.
